# Artificial Intelligence in Research: A Double-Edged Sword in Evidence Generation

**DOI:** 10.34172/aim.34419

**Published:** 2025-07-01

**Authors:** Seyyed Muhammad Mahdi Mahdavinoor, Seyyed Hatam Mahdavinoor

**Affiliations:** ^1^Student Research Committee, Faculty of Allied Medical Sciences, Mazandaran University of Medical Sciences, Sari, Iran; ^2^Department of Islamic Theology, Yadegar-e-Imam Khomeini (Rah) Shahre-rey Branch, Islamic Azad University, Tehran, Iran

## Introduction

 Artificial intelligence (AI) is rapidly integrating into research landscape, promising to revolutionize the way studies are conducted. From accelerating literature reviews to analyzing complex datasets, AI has the potential to significantly enhance the efficiency of core research activities. AI tools have been quickly embraced by researchers across various disciplines. Just a few months after the introduction of some of these tools, they were even credited as co-authors in several scientific articles and preprints.

 However, these powerful tools also introduce new challenges to research integrity. The dual nature of AI—its potential to either strengthen or undermine the quality of evidence—is becoming increasingly recognized. Enthusiasm for AI’s capabilities must be tempered with caution as uninformed or improper application may erode the foundation of evidence upon which clinical practice relies. Although AI offers substantial benefits in generating and synthesizing evidence, it can equally compromise research quality if researchers are not adequately informed about when, how, and where its use is appropriate.

 One of the less-discussed concerns is AI’s inability to accurately grasp the logic of citation. For instance, its failure to distinguish between primary and secondary sources can lead to distortion in scientific communication. Perhaps one of the most overlooked yet high-risk challenges of AI-based research tools is the current citation crisis. Scientific writing relies on a precise and trustworthy chain of sources and references; however, AI systems face serious issues in this area.

 Growing evidence suggests that chatbots may fabricate references—citations that appear to be legitimate but actually point to articles and journals that do not exist. In one study, when ChatGPT-3.5 was asked to generate scientific content with references, more than 55% of the sources it provided were entirely fabricated. Although newer models like GPT-4 have shown improved performance, they still produce fictitious references in approximately 18% of cases.^1^ Even when the references are real, AI models often struggle to differentiate between primary and secondary sources. For instance, a language model could cite a review article that discusses a discovery, while failing to reference the original primary source that first reported it. This type of misattribution violates one of the fundamental principles of scholarly writing: authors should, whenever possible, cite the original source of the findings.^2^ As previously emphasized, researchers are obligated to trace and cite primary sources, resorting to secondary citations only when access to the original ones is genuinely unavailable; even then, the indirect nature of the citation must be explicitly acknowledged using phrases like “as cited in”.^2^ Unfortunately, AI tools lack the discernment and judgment required for such decisions. They generate references purely based on patterns in their training data without any understanding of the credibility or actual relevance of the sources. As a result, researchers who rely on AI-generated bibliographies without careful verification may unintentionally fill their manuscript with fabricated sources or incorrect citations. This not only jeopardizes the integrity of the individual work but, if widespread, threatens to pollute the scientific literature with unreliable and unverifiable citations.

 Another previously noted concern is the generation of seemingly novel ideas by AI that are, in fact, not new and have already been published, while AI presents them without attribution as if they were original.^3^ Determining whether an idea is truly novel or merely a repetition can be challenging, even with thorough review by experienced researchers. One possible approach is to input the idea into an AI tool and ask it to identify similar existing concepts; however, even this method cannot guarantee that the idea has not been already claimed by someone else. It is therefore recommended that researchers avoid using AI to generate new research ideas until a reliable solution to this issue is found. While AI may potentially contribute to scientific progress by generating novel concepts, adherence to ethical principles is of paramount importance and must not be compromised.

 Therefore, the “citation crisis” requires serious attention; this is a technical flaw in AI tools that directly threatens the foundations of evidence-based science. Researchers must treat any AI-generated content merely as an initial draft requiring human verification, not as a final or reliable product. Only in this way can the scientific community benefit from the accelerating power of AI while simultaneously guarding against its potential harms.

 Like any other powerful technology, AI in research is a double-edged sword: AI can reduce inefficiencies, but if misused, it can harm the integrity of the scientific system. Indeed, the researchers are solely responsible for choosing how to use this tool. No scientific innovation is inherently good or bad; it is our usage mode that determines its consequences.^4^ With cautious implementation and robust oversight, the role of AI in research can be transformative, but in the absence of such frameworks, it may jeopardize the very foundation of evidence on which both evidence-based medicine and science as a whole rely.
